# Chromosomal Instability at Fragile Sites in Blue Foxes, Silver Foxes, and Their Interspecific Hybrids

**DOI:** 10.3390/ani11061743

**Published:** 2021-06-11

**Authors:** Marta Kuchta-Gładysz, Ewa Wójcik, Anna Grzesiakowska, Katarzyna Rymuza, Olga Szeleszczuk

**Affiliations:** 1 Department of Animal Reproduction, Anatomy and Genomics, Faculty of Animal Sciences, University of Agriculture in Krakow, ul. Mickiewicza 24/28, 30-059 Kraków, Poland; marta.kuchta-gladysz@urk.edu.pl (M.K.-G.); anna.grzesiakowska@urk.edu.pl (A.G.); olga.szeleszczuk@urk.edu.pl (O.S.); 2 Institute of Animal Science and Fisheries, Faculty of Agrobioengineering and Animal Husbandry, Siedlce University of Natural Sciences and Humanities, ul. Prusa 14, 08-110 Siedlce, Poland; 3 Institute of Agriculture and Horticulture, Faculty of Agrobioengineering and Animal Husbandry, Siedlce University of Natural Sciences and Humanities, ul. Prusa 14, 08-110 Siedlce, Poland; katarzyna.rymuza@uph.edu.pl

**Keywords:** fox, chromosomal instability, fragile site, B chromosome

## Abstract

**Simple Summary:**

The paper describes the karyotypes of blue and silver foxes and their hybrids, in terms of the numbers of A and B chromosomes and the frequency of fragile sites on chromosomes. Genome stability in these species is affected by Robertson translocations in the karyotype of the blue fox and by B chromosomes in the silver fox. The fragile sites assay was used as a biomarker to assess genome stability in foxes. This test enables the identification of breaks, chromatid gaps, and deletions. In healthy individuals, the number of these instabilities remains low. The test can be used to select individuals with the most stable genome for breeding of blue and silver foxes. The fewer an individual’s susceptible sites, the more likely it is to have good reproductive performance. This factor is extremely important in the case of blue foxes, which are an endangered species.

**Abstract:**

A cytogenetic assay based on fragile sites (FS) enables the identification of breaks, chromatid gaps, and deletions. In healthy individuals, the number of these instabilities remains low. Genome stability in these species is affected by Robertsonian translocations in the karyotype of the blue fox and by B chromosomes in the silver fox. The aims of the study were to characterise the karyotype of blue foxes, silver foxes, and their hybrids and to identify chromosomal fragile sites used to evaluate genome stability. The diploid number of A chromosomes in blue foxes ranged from 48 to 50, while the number of B chromosomes in silver foxes varied from one to four, with a constant number of A chromosomes (2n = 34). In interspecific hybrids, both types of karyotypic variation were identified, with the diploid number of A chromosomes ranging from 40 to 44 and the number of B chromosomes varying from 0 to 3. The mean frequency of FS in foxes was 4.06 ± 0.19: 4.61 ± 0.37 in blue foxes, 3.46 ± 0.28 in silver foxes, and 4.12 ± 0.22 in hybrids. A relationship was identified between an increased number of A chromosomes in the karyotype of the hybrids and the frequency of chromosomal breaks. The FS assay was used as a biomarker for the evaluation of genomic stability in the animals in the study.

## 1. Introduction

Genetic material contained in the cell nucleus, and organised into chromosomes, can be affected by damage or alterations, both structural and numerical, due to the effects of mutagenic factors [1,2]. One of the structural aberrations found in the karyotype of farm animals is centric fusion, also referred to as Robertsonian translocation. This phenomenon, which affects the karyotype of an individual, induces a change in the diploid number of chromosomes, which frequently results in abnormal chromosome segregation during division of both somatic and reproductive cells [3,4]. Centric fusion has been described in a number of animal species, including the polar fox (*Alopex lagopus*). Three karyotypic forms can be distinguished in this species on the basis of centric fusion: Two resulting from a Robertsonian translocation (2n = 48 and 2n = 49) and one without translocation (2n = 50 chromosomes) [5]. Numerical aberrations primarily affect the basic set of chromosomes (A chromosomes) in a given species/individual. A characteristic feature of the karyotype of the red fox (*Vulpes vulpes*) is the presence of additional supernumerary chromosomes referred to as B chromosomes [6,7,8,9]. The presence of B chromosomes in a karyotype is often an effect of evolutionary modifications in a species, and thus, changes in the number of chromosomes within a given family. According to Camacho et al. [10], B chromosomes lead to disturbances and abnormal segregation of chromosomes, mainly A chromosomes, during cell division. Interspecific crossing results in new genetic variation, but also to mixing or accumulation of aberrations occurring in the parental species. Crosses between *Alopex lagopus* and *Vulpes vulpes* are bred, in order to improve their functional characteristics and obtain materials desired by the consumer: High-quality, short but voluminous fur, with a structure resembling blue fox fur, but the colour of silver fox fur. In addition, the crosses have a relatively large body size, like silver foxes, which additionally enhances their production value [11,12,13]. 

Cytogenetic assays can be used to determine both numerical and structural changes, and to assess the susceptibility of chromosomes to damage [1,14]. Defects in single and double strands of DNA generate chromosomal instabilities, including chromosomal aberrations and increased fragility [1,15]. Highly sensitive cytogenetic assays, such as those based on fragile sites (FS), are used to identify various types of damage, including gaps and chromatid breaks in chromosomes [16]. Damage of this type may occur at sites where two-, three- or four-nucleotide repeats accumulate. These sequences affect the dynamics of replication. The weakening of links between nucleosomes leads to decondensation of genetic material and generates damage [17]. FS can also occur as AT-rich nucleotide sequences, which do not contain repeat sequences or show a tendency to expand [18,19]. Their structure has a distinct arrangement in the form of islands. This increases the flexibility of the sequences, leading to the formation of secondary structures that disrupt the processes of replication, transcription and chromatin organization [17,20]. Fragile sites may result from malfunctioning mechanisms that correct disruptions in the progress of replication forks or from malfunctioning replication and transcription mechanisms [21,22]. Fragile sites are an integral part of chromosome structure, and their occurrence in the genome is spontaneous [23,24]. In healthy individuals their number remains low, while any abnormalities cause their level to increase significantly. FS analysis is possible when cells are cultured under specific conditions, which favour the inhibition of replication. This results in the expression of fracture sites manifested as gaps, splits, breaks, and deletions on chromosomes.

The aims of this study were to characterise the karyotype of blue foxes, silver foxes, and their interspecific hybrids in terms of the number of chromosomes and to identify chromosomal fragile sites.

## 2. Materials and Methods

The study was performed with 36 farmed foxes: Blue foxes (*Alopex lagopus*), silver foxes (*Vulpes vulpes*), and their interspecific hybrids (*Alopex lagopus* × *Vulpes vulpes*). An experimental group of 12 animals, 6 males (M) and 6 females (F), was established for each species. The age of the animals was between 9 to 10 months.

Cells obtained from an in vitro culture of peripheral whole blood lymphocytes, collected from the *v*. *cephalica antebrachi* of one-old year foxes, were used in the study. Samples of whole peripheral blood were obtained during routine veterinary examination. Cell cultures were grown under standard conditions (in vitro culture time 72 h; temperature 37.5 °C; 5% CO, with stable humidity). At 65 h of incubation, the culture medium was supplemented with 5 ug mL^−1^ of BrdU (5-bromodeoxyuridine, Sigma Aldrich, Darmstadt, Germany). As a hypotonic solution we used 0.65% potassium chloride (Sigma Aldrich). The cells were fixed with Carnoy fixative. The material was used to prepare suspensions, which were subsequently stained with Hoechst 33258 in 2 × SSC (0.75M sodium chloride + 0.075 sodium citrate both Sigma Aldrich) with simultaneous exposure to UV radiation (UV-C lamp 185–254 nm, 15W G15; distance 60 cm, Philips, Amsterdam, The Netherlands). Next, they were incubated in 2 × SSC at 65 °C, stained with 4% Giemsa (Sigma Aldrich), and dried at 40 °C. Fifty metaphases per animal were analyzed. The parameter tested was the number of identified chromosomal instabilities in the form of chromatid gaps, breaks, and deletions in chromosomes. Blue and silver foxes and their interspecific hybrids were analysed to determine karyotype variability with respect to the number of A and B chromosomes. The number of FS instabilities in karyotypes with constant and variable numbers of A chromosomes and in karyotypes with B chromosomes was determined. 

Microscopic analyses and photographic documentation were performed using a Zeiss Imager A2 epifluorescence microscope (Carl Zeiss, Oberkochen, Germany) fitted with a Zeiss AxioCam MRc5 camera (Carl Zeiss). The Statistica 12.0 software package (StatSoft Inc., Kraków, Poland) was used for statistical data processing. One-way ANOVA, with the fox species as the factor tested, was used to compare the number of gaps, breaks, deletions, and fragile sites found in the cells of different species. Prior to performing the calculations, the normal distribution of characteristics was assessed using the chi-square test. Logarithmic data transformation was applied for the characteristics in which distributions were inconsistent with normal distribution. Student’s *t*-test was used to compare the number of gaps, breaks, deletions, and FS between males and females within each species. The relationship between different karyotypes of the species and the appearance of various FS instabilities was evaluated. Pearson’s correlation analysis was performed for a significance level of *p* ≤ 0.05 to analyze the relationships between the characteristics.

All experiments were conducted in accordance with the recommendations in Directive 63/2010/EU and the Journal of Laws of the Republic of Poland of 2015 on the protection of animals used for scientific or educational purposes. The study was approved by the Polish Laboratory Animal Science Association (nos. 3235/2015; 4466/2017; 17/2010; 1/2020).

## 3. Results

The numbers of A and B chromosomes in the karyotypes of the animals (blue foxes, silver foxes, and interspecific hybrids) are shown in Table 1. Variability in the number of chromosomes due to karyotype polymorphism was detected. In blue foxes, the number of A chromosomes ranged from 2n = 48 to 2n = 50, but the most common diploid number of chromosomes was 2n = 49. In silver foxes, in addition to a constant number of A chromosomes in the 2n = 34 + B karyotype, a variable number of B chromosomes, ranging from 1 to 4, was identified. The most commonly observed number was 2 B. In interspecific hybrids, both types of karyotype variation were detected. The number of A chromosomes in the cells varied from 2n = 40 + B to 2n = 44 + B, while the number of B chromosomes ranged from 0 to 3. The most prevalent karyotype was 2n = 42, and the most common number of B chromosomes was 2 (Table 1). 

**Figure 1:**
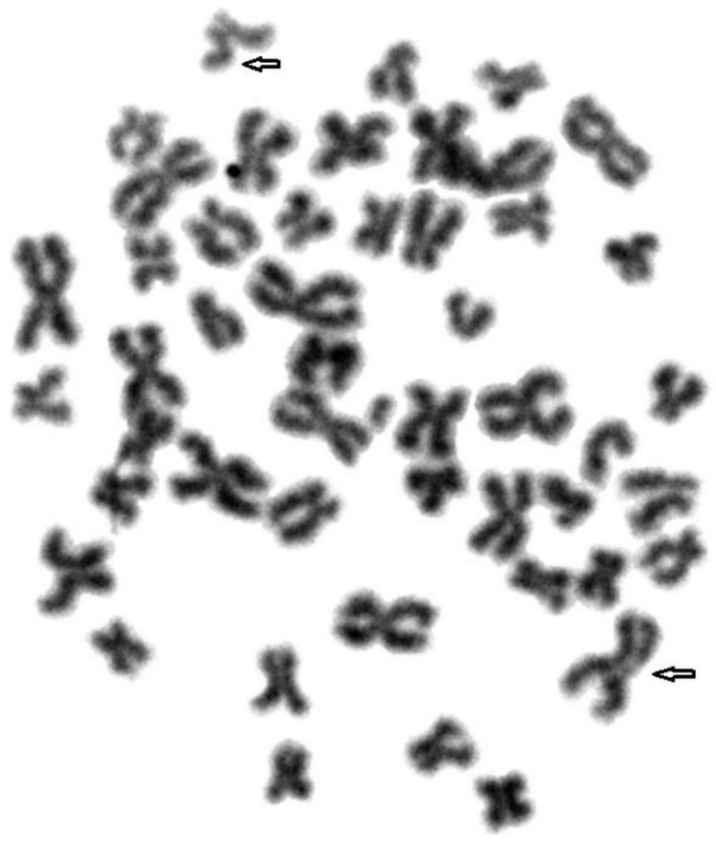
Fragile sites in the chromosomes of blue foxes. Damage marked with arrows.

**Figure 2:**
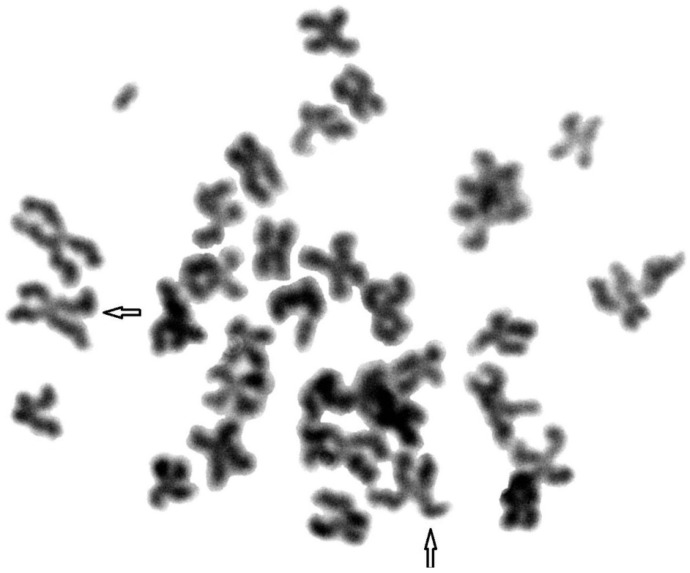
Fragile sites in the chromosomes of silver foxes. Damage marked with arrows.

**Figure 3:**
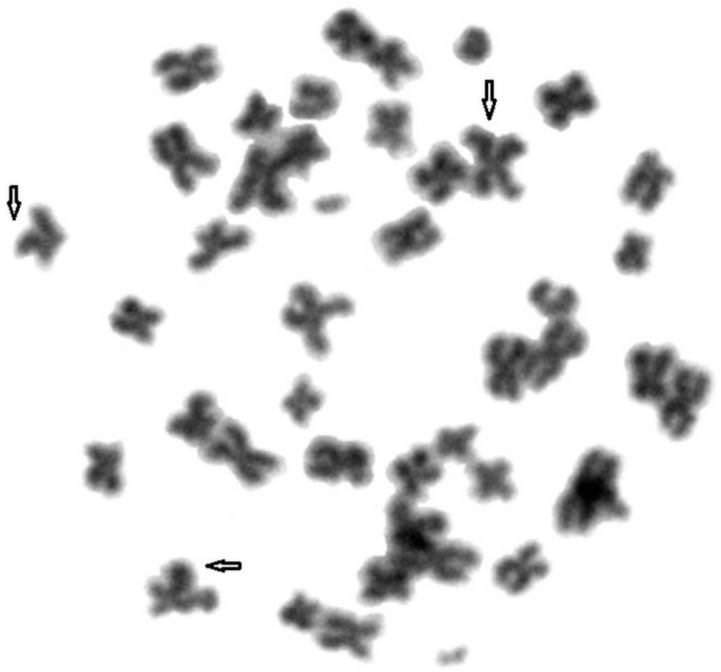
Fragile sites in the chromosomes of hybrids. Damage marked with arrows.

To compare genome stability in blue and silver foxes and their hybrids, fragile sites were identified in their chromosomes. A total of 1800 metaphase plates were analysed. Figure 1, Figure 2 and Figure 3 show FS damage identified in (1) blue foxes, (2) silver foxes, and (3) hybrids. Table 2 lists the FS instabilities identified (gaps, breaks, and deletions) arranged by species.

The mean frequency of FS in the animals was 4.06 ± 0.19. The differences were identified in the frequency of FS between species. However, statistically significant differences were only observed between the parental species (*p* = 0.000); no statistically significant differences were noted between blue foxes and hybrids (*p* = 0.071) or between silver foxes and hybrids (*p* = 0.068) (Table 2). Statistically significant differences were noted between silver foxes and hybrids in the frequency of gap formation (*p* = 0.00), but no statistically significant differences were found between blue foxes and silver foxes (*p* = 0.31) or between blue foxes and hybrids (*p* = 0.28). Statistically significant differences were found between blue foxes and silver foxes and between blue foxes and hybrids in the frequency of break formation (*p* = 0.000; *p* = 0.00), but not between silver foxes and hybrids (*p* = 0.41). Statistically significant differences were also noted between blue foxes and hybrids in the case of deletions (*p* = 0.00), but not between the parental species (*p* = 0.42) or between silver foxes and hybrids (*p* = 0.12). FS forms detected in the fox species were dominated by breaks, while deletions were the least common type (Table 3). The mean FS frequency was 4.17 ± 0.42 in male foxes and 3.95 ± 0.63 in females. There were no statistically significant differences between males and females. Similarly, no statistically significant differences were seen between males and females within species (blue *p* = 0.31; silver *p* = 0.24; hybrid *p* = 0.28) (Table 2) or in the analysis of different forms of instabilities: gaps (blue *p* = 0.33; silver *p* = 0.22; hybrid *p* = 0.11), breaks (blue *p* = 0.42; silver *p* = 0.68; hybrid *p* = 0.61), and deletions (blue *p* = 0.64; silver *p* = 0.31; hybrid *p* = 0.74) (Table 4). 

The relationships between both forms of polymorphic karyotype in the fox species and the appearance of FS instabilities were evaluated. The frequency of FS was not shown to be correlated with the variable number of A chromosomes in blue foxes or with the variable number of B chromosomes in silver foxes. In interspecific hybrids with a variable number of A and B chromosomes in the karyotype, a correlation was detected between A chromosomes and the appearance of chromosomal breaks (r = 0.49). The more A chromosomes were observed in the karyotype, the more fragile sites were noted.

## 4. Discussion

Fragile sites are a visual effect of damage to chromatid structure, which are induced by adverse factors. The estimation of FS frequency can serve as a biomarker for evaluating animal health and genome stability. A large number of fragile sites negatively affects multiple processes in an animal, causing impairment of bodily functions. Cytogenetically, FS are sites where the replication process was disrupted and the resulting errors were not corrected by repair mechanisms, or were repaired incorrectly. FS have retained evolutionary conservatism in all animals [25,26,27,28]. The number of spontaneous fragile sites varies from 1 to 10 in young, healthy animals which are not exposed to any negative mutagenic or carcinogenic environmental factors. The appearance of FS is also determined by the species of animal [16,29,30]. Our study included a detailed analysis of generated FS types. The most common form of FS was found to be breaks, followed by gaps and deletions. In contrast, Wójcik et al. [31], studying FS in quails, observed more chromosomal gaps than breaks. It is likely that the prevalence of various types of chromosomal damage can also depend on the species of animal. 

In addition to the factors mentioned above, FS frequency is also linked to the age of animals. Various FS frequencies were observed by Ali et al. [26] in sheep karyotypes, by Wójcik and Szostek [16] in cows, and by Wójcik and Sokół [29] in pigs. The younger the animals were, the less chromosomal damage was observed. This pattern was observed in our study as well. The low frequency of fragile sites observed in our study supports the claim that young animals have a stable genome. Consequently, age should be recognised as a key factor determining genome instability. Changes accompanying the cell ageing process contribute to impairment of cellular mechanisms: Replication, control, and repair. Consequently, younger animals are more able to cope with negative factors causing damage to genetic material and impairment of cellular mechanisms. In this way, errors are eliminated, resulting in a low level of instability. Prolonged exposure of the genome to an unfavourable environment increases chromosomal fragility [32,33]. Many authors have found that the sex of animals has no effect on FS instabilities, observing very similar means for males and females and no statistically significant differences [26,31,34,35]. The differences observed between males and females in our study were also minor and statistically non-significant. 

Our study also verified whether two different polymorphic karyotype characteristics affect the frequency of FS. Blue foxes had a variable number of chromosomes in their basic karyotype, known as A chromosomes (2n = 48/49/50), while silver foxes had a constant number of these chromosomes, but a variable number of B chromosomes (1–3). Makinen and Gustavsson [11], Graphodatsky et al. [36], Świtoński et al. [8], and Grzesiakowska et al. [37] found that the number of A chromosomes in polar foxes was 2n = 48 to 50. However, the heterozygous karyotype (2n = 49) was more prevalent. This form of karyotype was observed in our study as well. The second polymorphic form found in silver foxes and associated with the presence of B chromosomes is rare in other mammals. This karyotype occurs in three species of the family Canidae: the red fox, Chinese raccoon dog, and Japanese raccoon dog [38,39]. The number of B chromosomes is variable, ranging from 0 to 10. Basheva et al. [40] found that the number of B chromosomes varied from 0 to 10, while Graphodatsky et al. [36] reported a range from 0 to 7, Grzesiakowska et al. [37] 0–4, and Świtoński et al. [8] 0–8. The most prevalent karyotypes contained 2 or 3 chromosomes. In our study, the number of these chromosomes ranged from 0 to 4, and the most prevalent karyotype form consisted of two B chromosomes. Interspecific hybrids are model animals used to compare closely related parental species with certain differences in genetic characteristics. The fox hybrids evaluated in our study combine the characteristics of their parental karyotypes: A variable number of both A and B chromosomes. The diploid number of A chromosomes ranged from 40 to 44 and was higher than in silver foxes but lower than in blue foxes. The number of B chromosomes was similar to the number found in silver foxes. Mäkinen and Gustavsson [11] reported the modal number of chromosomes to be 2n = 41 + (1–3) or 2n = 42 + (1–3), while Bugno-Poniewierska et al. [13] found that 2n = 40, 2n = 41 or 2n = 42, with the number of B chromosomes varying from 0 to 4. 

The FS frequencies observed in hybrids (except for breaks in blue foxes) were higher than in their parents, and interestingly, their number increased with the number of A chromosomes. Silver foxes had a more stable genome than blue foxes and hybrids. Vaneste et al. [41], Alfarawati et al. [42], Kozubska-Sobicińska, and Danielak-Czech [43] attribute reproductive failure to Robertsonian translocation, among other factors. Although the animals have a normal phenotype, they are affected by impaired fertility, oestrous cycle disturbances, and an extended inter-birth interval. In carriers of centric fusions, meiosis is disrupted. In our study, the most common form of karyotype found in blue foxes had 49 diploid chromosomes. This is known as a heterozygous karyotype. Animals with this type of karyotype are characterised by impaired reproductive performance [5,8,37,44]. Infertility in hybrids may result from the accumulation of specific karyotypes containing different numbers of A chromosomes, as well as from the presence of B chromosomes, which are centric fragments remaining after Robertsonian translocation. Their number is variable due to their unstable behaviour during cell divisions and a non-Mendelian pattern of inheritance. They have a tendency to form multivalent and univalent configurations during meiosis prophase I. Unfortunately, both forms of chromosomal instability, as well as an increased frequency of FS, are recognised as potential additional contributors to reproductive challenges. Chromosome fragility causes abnormalities in the karyotype of gametes and subsequently embryos, potentially leading to spontaneous abortion or genetic defects in the offspring. Fragile site analysis is an important diagnostic tool in animal breeding and reproduction [45,46,47,48]. Interspecific hybrids are either sterile or characterised by impaired reproductive parameters and hybrid depression. Mechanisms of reproductive isolation, both pre- and postzygotic, prevent the formation of zygotes. The problem not only arises in the act of mating itself, due to the mismatch of reproductive organs, and in mating simulation, but also manifests in gametic incompatibility [49,50]. Consequently, crosses between blue foxes and silver foxes are obtained via artificial insemination. Following the fusion of gametes, the embryo is implanted. However, the postzygotic barrier consisting in the functional incompatibility of sexual communication genes and their interactions prevents the development of fertile animals [51]. Furthermore, various polymorphic features observed in the karyotype, as well as differences in the morphology and structure of chromosomes between species contribute to the infertility and mortality of hybrids [52]. 

## 5. Conclusions

The specific karyotypes of blue and silver foxes make them an interesting subject of study. Their hybrids are model animals which have the karyotype form of both parents, i.e., a variable number of A and B chromosomes. In our study, the most common karyotype form in blue foxes was the heterozygous karyotype. Its presence, as well as the presence of translocation chromosomes and the higher FS frequency identified in this species, suggest that this might be the cause of the impaired reproductive performance observed in these foxes. The hybrids, as the F1 generation, had karyotype features inherited from their parents, with chromosomes from both blue and silver foxes—translocation chromosomes and B chromosomes. In addition, they were shown to have a high frequency of FS, which may explain their infertility. Silver foxes had the lowest frequency of FS-type instabilities, so the species was considered to have a more stable genome than the other animals.

## Figures and Tables

**Table 1:** Numbers of A and B chromosomes in the karyotypes of blue foxes, silver foxes, and their hybrids.

Foxes					
Blue		Silver		Hybrid	
Chromosomes					
A	B	A	B	A	B
49	0	34	3	42	2
48	0	34	2	41	2
48	0	34	2	41	2
48	0	34	2	41	2
48	0	34	3	42	1
49	0	34	4	40	1
49	0	34	2	40	3
49	0	34	2	44	1
49	0	34	1	42	1
50	0	34	1	42	2
50	0	34	1	40	3
49	0	34	1	42	1

**Table 2:** Number of FS identified in each fox species.

Sex	Foxes		
	Blue	Silver	Hybrid
Male	4.80 ^a^ ± 0.14	3.42 ^a^ ± 0.28	4.28 ^a^ ± 0.26
Female	4.41 ^a^ ± 0.52	3.49 ^a^ ± 0.30	3.95 ^a^ ± 0.19
Mean	4.61 ^a^ ± 0.37	3.46 ^b^ ± 0.28	4.12 ^ab^ ± 0.22

Means designated with different letters vary significantly within a sex at *p* ≤ 0.05 (mean ± standard deviation).

**Table 3:** Number of FS—types of damage.

Foxes	Damage (Number per Cell)		
	Gaps	Breaks	Deletions
Blue	0.51 ^ab^ ± 0.17	3.9 1 ^a^ ± 1.09	0.19 ^b^ ± 0.13
Silver	0.46 ^b^ ± 0.21	2.76 ^b^ ± 0.81	0.23 ^ab^ ± 0.13
Hybrid	0.74 ^a^ ± 0.40	3.03 ^b^ ± 0.53	0.34 ^a^ ± 0.12

Means designated with different letters differ significantly within a sex (*p* ≤ 0.05).

**Table 4:** Number of FS—comparison between males and females.

Foxes	Sex	Damage (Number per Cell)		
		Gaps	Breaks	Deletions
Blue	Male	0.54 ^a^ ± 0.16	4.10 ^a^ ± 0.45	0.16 ^a^ ± 0.04
	Female	0.47 ^a^ ± 0.18	3.72 ^a^ ± 1.52	0.22 ^a^ ± 0.18
Silver	Male	0.32 ^a^ ± 0.12	2.87 ^a^ ± 0.85	0.23 ^a^ ± 0.11
	Female	0.60 ^a^ ± 0.18	2.65 ^a^ ± 0.85	0.24 ^a^ ± 0.16
Hybrid	Male	0.89 ^a^ ± 0.47	3.10 ^a^ ± 0.39	0.29 ^a^ ± 0.07
	Female	0.58 ^a^ ± 0.27	2.97 ^a^ ± 0.68	0.40 ^a^ ± 0.14

Means designated by different letters vary significantly within sex (*p* ≤ 0.05).

## Data Availability

This study did not report any data.
